# Opioid Receptors in Immune and Glial Cells—Implications for Pain Control

**DOI:** 10.3389/fimmu.2020.00300

**Published:** 2020-03-04

**Authors:** Halina Machelska, Melih Ö. Celik

**Affiliations:** Department of Experimental Anesthesiology, Charité – Universitätsmedizin Berlin, Corporate Member of Freie Universität Berlin, Humboldt-Universität zu Berlin, Berlin Institute of Health, Berlin, Germany

**Keywords:** analgesia, astrocytes, microglia, nociceptin/orphanin FQ, oligodendrocytes, opioid-induced hyperalgesia, opioid peptides, opioid receptor signaling

## Abstract

Opioid receptors comprise μ (MOP), δ (DOP), κ (KOP), and nociceptin/orphanin FQ (NOP) receptors. Opioids are agonists of MOP, DOP, and KOP receptors, whereas nociceptin/orphanin FQ (N/OFQ) is an agonist of NOP receptors. Activation of all four opioid receptors in neurons can induce analgesia in animal models, but the most clinically relevant are MOP receptor agonists (e.g., morphine, fentanyl). Opioids can also affect the function of immune cells, and their actions in relation to immunosuppression and infections have been widely discussed. Here, we analyze the expression and the role of opioid receptors in peripheral immune cells and glia in the modulation of pain. All four opioid receptors have been identified at the mRNA and protein levels in immune cells (lymphocytes, granulocytes, monocytes, macrophages) in humans, rhesus monkeys, rats or mice. Activation of leukocyte MOP, DOP, and KOP receptors was recently reported to attenuate pain after nerve injury in mice. This involved intracellular Ca^2+^-regulated release of opioid peptides from immune cells, which subsequently activated MOP, DOP, and KOP receptors on peripheral neurons. There is no evidence of pain modulation by leukocyte NOP receptors. More good quality studies are needed to verify the presence of DOP, KOP, and NOP receptors in native glia. Although still questioned, MOP receptors might be expressed in brain or spinal cord microglia and astrocytes in humans, mice, and rats. Morphine acting at spinal cord microglia is often reported to induce hyperalgesia in rodents. However, most studies used animals without pathological pain and/or unconventional paradigms (e.g., high or ultra-low doses, pain assessment after abrupt discontinuation of chronic morphine treatment). Therefore, the opioid-induced hyperalgesia can be viewed in the context of dependence/withdrawal rather than pain management, in line with clinical reports. There is convincing evidence of analgesic effects mediated by immune cell-derived opioid peptides in animal models and in humans. Together, MOP, DOP, and KOP receptors, and opioid peptides in immune cells can ameliorate pathological pain. The relevance of NOP receptors and N/OFQ in leukocytes, and of all opioid receptors, opioid peptides and N/OFQ in native glia for pain control is yet to be clarified.

## Introduction

Opioid receptors comprise four members, the classical μ (MOP), δ (DOP), and κ (KOP) receptors, and the non-classical nociceptin/orphanin FQ (NOP) receptor [reviewed by (1)] (Table 1). They belong to the superfamily of seven transmembrane domain, G protein-coupled receptors, are encoded by the four respective genes [(2–5), reviewed by (6)], and their structures have been cleared by crystal analysis (7–10). The classical opioid receptors are sensitive to the antagonist naloxone and their endogenous agonists are opioid peptides, such as β-endorphin, enkephalins (Met-, Leu-enkephalin), and dynorphins (dynorphin A, B, α-neoendorphin). β-endorphin and enkephalins bind MOP and DOP receptors, whereas dynorphin A 1-17 preferentially acts at KOP receptors. Opioid peptides derive from the respective precursors, proopiomelanocortin (POMC) (11, 12), proenkephalin (PENK) (13, 14), and prodynorphin (PDYN) (15–17). Endomorphins (endomorphin-1,−2) are additional, putative endogenous opioid peptides with high selectivity at MOP receptors (18); their precursor has not yet been identified [reviewed by (19)]. NOP receptors are insensitive to antagonism by naloxone, have low affinity for opioid peptides, and their selective endogenous agonist is nociceptin/orphanin FQ (N/OFQ), which derives from prepro-N/OFQ (ppN/OFQ) [(20, 21) reviewed by (6)] (Table 1).

**Table 1:** Characterization of the opioid system.

**Opioid receptor**	**Endogenous agonists**		Exogenous ligands^*^		**Effects on pain**	**Side effects**
	**Precursor**	**Peptide**	**Agonists**	**Antagonists**		
MOP	POMC Not identified	END^#^ EM-1 EM-2	Morphine Fentanyl Oxycodone Methadone DAMGO	Naloxone Naltrexone CTAP CTOP β-FNA	Analgesia	Respiratory depression, sedation, constipation, nausea, vomiting, reward/euphoria, dependence/withdrawal
DOP	PENK	ENKs^#^	DPDPE DELTs SNC80	Naloxone Naltrexone Naltrindole ICI 174,864	Analgesia	Convulsions, reward
KOP	PDYN	DYNs	Bremazocine U50,488 U69,593	Naloxone Naltrexone NorBNI	Analgesia	Aversion/dysphoria, sedation, diuresis, psychotomimesis (abnormal perception of space, time and visual experience, self-control loss, depersonalization)
NOP	ppN/OFQ	N/OFQ	Ro 64-6198 SCH 221510	J-113397 SB-612111	Analgesia Hyperalgesia/anti-opioid action (brain)	Sedation, constipation, diuresis, hypotension, bradycardia, learning and memory impairment, motor disturbance

* *Listed are selected ligands most often used in humans or tested in animals*.

# *END also binds DOP receptors; ENKs also bind MOP receptors*.

*β-FNA, β-funaltrexamine; CTAP, D-Phe-Cys-Tyr-D-Trp-Arg-Thr-Pen-Thr-NH_2_; CTOP, D-Phe-Cys-Tyr-D-Trp-Orn-Thr-Pen-Thr-NH_2_; DAMGO, [D-Ala^2^,N-Me-Phe^4^,Gly^5^-ol]-enkephalin; DELTs, deltrophins (deltorphin I, II); DPDPE, D-Pen^2^, D-Pen^5^-enkpephalin; DYNs, dynorphins (dynorphin A, B, α-neoendorphin); EM, endomorphin; END, β-endorphin; ENKs, enkephalins (Met-, Leu-enkephalin); ICI 174,864, N,N-diallyl-Tyr-Aib-Aib-Phe-Leu; J-113397, 1-[(3R,4R)-1-cyclooctylmethyl-3-hydroxymethyl-4-piperidyl]-3-ethyl-1,3-dihydro-2H-benzimidazol-2-one; N/OFQ, nociceptin/orphanin FQ; NorBNI, norbinaltorphimine; PDYN, prodynorphin; PENK, proenkephalin; POMC, proopiomelanocortin; ppN/OFQ, prepro-nociceptin/orphanin FQ; Ro 64-6198, (1S,3aS)-8-(2,3,3a,4,5,6-hexahydro-1H-phenalen-1-yl)-1-phenyl-1,3,8-triaza-spiro[4.5]decan-4-one; SB-612111, (2)-cis-1-methyl-7-[[4-(2,6-dichlorophenyl)piperidin-1-yl]methyl]-6,7,8,9-tetrahydro-5Hbenzocyclohepten-5-ol; SCH 221510, [3-endo-8-[bis(2-methylphenyl)methyl]-3-phenyl-8-azabicyclo [3.2.1]octan-3-ol]; SNC80, 4-(alpha-(4-allyl-2,5-dimethyl-1-piperazinyl)-3-methoxybenzyl)-N,N-diethylbenzamide; U50,488, trans-(±)3,4-dichloro-N-methyl-N-[2-(1-pyrrolidinyl)-cyclohexyl]-benzeneacetamide; U69,593, (+)-(5α,7α,8β)-N-Methyl-N-[7-(1-pyrrolidinyl)-1-oxaspiro[4.5]dec-8-yl]-benzeneacetamide*.

**Figure 1:**
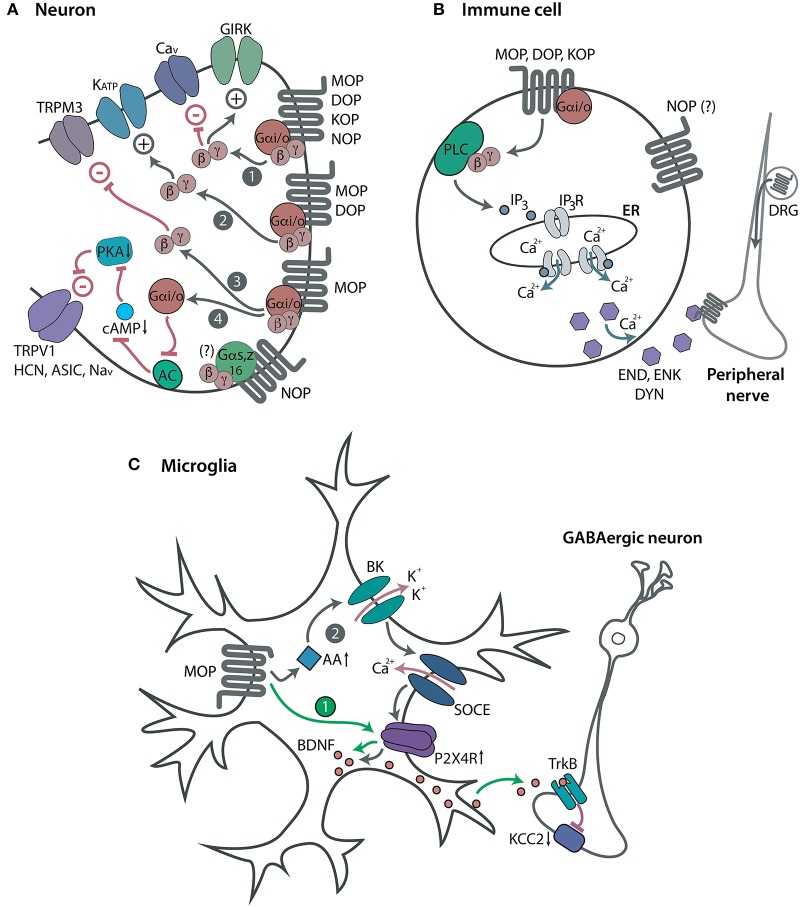
Opioid receptors and modulation of pain. **(A)** Neuronal opioid receptors. Acute activation of Gαi/o-coupled MOP, DOP, KOP, and NOP receptors in central or peripheral sensory neurons leads to the opening of GIRK channels and closing of Ca_v_ channels via the Gβγ (path 1). Through the Gβγ, MOP and DOP receptors also open K_ATP_ channels (path 2), and MOP receptors close TRPM3 channels (path 3). Through the Gαi/o, MOP receptors inhibit AC, cAMP formation and PKA activity, which leads to closing of TRPV1, HCN, ASIC, and Na_v_ channels (path 4). All these effects decrease neuronal excitability, which results in analgesia. NOP receptors also couple to Gαs, Gαz or Gα16, but their role in pain modulation is unknown (indicated by a question mark). **(B)** Immune cell opioid receptors. Acute activation of Gαi/o-coupled MOP, DOP, and KOP receptors in immune cells accumulating in peripheral injured tissue leads to the Gβγ-mediated activation of PLC and production of IP_3_ which activates IP_3_R in endoplasmic reticulum (ER). This results in the intracellular Ca^2+^-dependent release of opioid peptides, β-endorphin (END), Met-enkephalin (ENK), and dynorphin A 1-17 (DYN). The secreted opioid peptides activate opioid receptors (MOP, DOP, KOP) in peripheral nerves and diminish pain. NOP receptors are also expressed in immune cells, but their function has not been identified (indicated by a question mark). **(C)** Microglial opioid receptors. Repetitive activation of MOP receptors in spinal cord microglia upregulates purinergic P2X4 receptors (P2X4R), which triggers the release of BDNF from microglia. The secreted BDNF activates the tropomyosin receptor kinase B (TrkB) to downregulate the K^+^-Cl^−^ co-transporter KCC2 in GABAergic spinal neurons, which leads to their disinhibition (path 1). Microglial MOP receptor activation can also elevate AA levels to facilitate the opening of BK channels. This triggers the Ca^2+^ influx via store-operated Ca^2+^ entry (SOCE) and consequent upregulation of P2X4R and BDNF synthesis in microglia (path 2). Both signaling pathways are suggested to potentiate the neurotransmission in the spinal cord and account for OIH. However, these effects may be a consequence of opioid withdrawal rather than direct hyperalgesic opioid actions. Expression and function of DOP, KOP, and NOP receptors in glia are yet to be clarified.

Neuronal opioid receptors are widely distributed throughout the peripheral (trigeminal and dorsal root ganglia) and central (spinal cord, brain) nervous system. All four opioid receptors mediate analgesia in animal models. However, the majority of clinically used opioids for pain treatment are MOP receptor agonists (e.g., morphine, fentanyl, oxycodone). Centrally acting KOP receptor agonists are of limited utility due to dysphoric and psychotomimetic effects (22–24), whereas DOP and NOP receptor agonists are not available for clinical use. Mechanistically, following acute activation by an agonist (endogenous or exogenous), opioid receptors couple to the pertussis toxin-sensitive heterotrimeric Gi/o proteins, which dissociate into Gαi/o and Gβγ subunits to interact with various intracellular effectors (Figure 1A). Activation of all four MOP, DOP, KOP, and NOP receptors can result in the Gαi/o-dependent inhibition of adenylyl cyclases (AC) and cyclic adenosine monophosphate (cAMP) formation [reviewed by (25, 26)]. However, the exact pathway in which these actions result in pain inhibition has only been described for MOP receptors. Hence, the decreased cAMP production leads to the inhibition of protein kinase A (PKA) activity, which results in the suppression of various ion channels involved in pain facilitation. These channels include the heat sensor transient receptor potential cation channel subfamily V member 1 (TRPV1), hyperpolarization-activated cyclic nucleotide-gated (HCN) channels, acid-sensing ion channels (ASIC), and voltage-gated Na^+^ (Na_v_) channels (27–31). Through the Gβγ, all four receptors close voltage-gated Ca^2+^ (Ca_v_) channels (32–34) and open G protein-coupled inwardly-rectifying K^+^ (GIRK or K_ir_3) channels (35–39), whereas MOP and DOP receptors also activate adenosine triphosphate-sensitive K^+^ (K_ATP_) channels [(40), reviewed by (26, 41)]. Additionally, MOP receptors inhibit heat-sensing transient receptor potential cation channel subfamily M member 3 (TRPM3) (42). These opioid receptor-mediated actions lead to the hyperpolarization and decreased excitability of central and peripheral sensory neurons, as well as to the diminished release of excitatory mediators from these neurons, including substance P (43–48), calcitonin gene-related peptide (45, 49–51), and glutamate (52, 53). In addition, activation of MOP receptors in the brain activates descending noradrenergic pathways, which leads to increased release of noradrenaline in the spinal cord (54, 55). All above described effects underlay the opioid receptor-induced analgesia [reviewed by (26, 41, 56–60)]. Additionally, the activation of NOP receptors in the brain can lead to hyperalgesia or anti-opioid actions in animal models (20, 21, 61). NOP receptors also couple to pertussis toxin-insensitive Gαs, Gαz, or Gα16 proteins (62, 63), but the role of these pathways in pain modulation is unknown.

Neuronal opioid receptors also mediate numerous side effects, such as respiratory depression, nausea, vomiting, reward/euphoria, dependence/withdrawal (MOP), convulsions (DOP), aversion/dysphoria, psychotomimesis (KOP), learning and memory impairment, motor disturbance, hypotension, bradycardia (NOP), sedation (MOP, KOP, NOP), constipation (MOP, NOP), and diuresis (KOP, NOP) [reviewed by (56, 57, 64–67)]. The main efforts are currently directed toward the development of novel ligands that exert analgesia with reduced side effects [reviewed by (57, 68, 69)].

Opioids (endogenous and exogenous) can also affect the function of immune cells, including proliferation, maturation, chemotaxis, trafficking, phagocytosis, cytokine, and chemokine receptor expression, cytokine synthesis and secretion. These effects were predominately assessed *in vitro* and the results were often contradictory, which depended on experimental conditions (e.g., cultured cell types, lines or clones, duration of cultures, media composition, doses and timing of opioid exposure) [reviewed by (70, 71)]. The immunomodulatory actions of opioids in the context of immunosuppression and infections have been widely reviewed (72–77).

In this article, we analyze the expression of opioid receptors in peripheral immune cells and glia, and discuss their contribution to the modulation of pain. Specifically, we address peripheral immune cells, such as lymphocytes, monocytes, macrophages and granulocytes in the blood and peripheral tissue. Although in pathological pain conditions some of these cells also infiltrate central nervous system (78–81), they have not been examined for the expression of opioid receptors. Glia represent immune cells of the nervous system and they include microglia, astrocytes and oligodendrocytes in the spinal cord and brain, satellite glial cells in trigeminal and dorsal root ganglia, and Schwann cells in peripheral nerves [reviewed by (82, 83)]. Of these cells, microglia, astrocytes and oligodendrocytes were so far tested for the presence of opioid receptors, and they are addressed in the following sections. Additionally, we describe the relevance of opioid peptides and N/OFQ derived from immune and glial cells to pain control.

## Expression of Opioid Receptors in Immune and Glial Cells

Expression and function of opioid receptors have been extensively examined *in vitro* using cell lines or cultured primary immune and glial cells. Since the results obtained in such conditions often vary with experimental setups, including cell origin and density (84–86), and do not reflect the *in vivo* situation, we focus on *ex vivo* studies which examined tissue or freshly isolated, not cultured primary immune and glial cells.

### Immune Cells

Expression of opioid receptors in peripheral immune cells has been postulated since the early 1980s [reviewed by (71, 76, 87)], but some findings still remain controversial. For example, some authors using various methods, such as radioligand binding, flow cytometry, polymerase chain reaction (PCR) and real-time quantitative PCR (qPCR) did not detect MOP, DOP, and KOP receptors in peripheral blood mononuclear cells (PBMC) or all blood cells from healthy human donors; only NOP receptor mRNA was found by PCR and qPCR (88, 89). Similarly, MOP and DOP mRNAs were not identified in healthy human blood lymphocytes or monocytes (90, 91), and MOP and KOP mRNAs were not found in mouse splenocytes and T lymphocytes (90) (Table 2).

**Table 2:** Studies that did not detect opioid receptors in native, not cultured immune and glial cells.

**Opioid receptor**	**mRNA, protein**	**Cell types**	**References**
MOP	mRNA	Human blood lymphocytes, PBMC or whole blood cells Mouse splenocytes, T lymphocytes Rat, mouse spinal cord microglia Rat spinal cord or nucleus accumbens astrocytes	(88, 89, 91) (90) (92, 93) (93, 94)
	Protein	Human PBMC Mouse spinal cord microglia Mouse spinal cord astrocytes	(88) (92, 95) (95)
DOP	mRNA	Human blood lymphocytes, monocytes Human PBMC, whole blood cells Rat nucleus accumbens microglia Rat nucleus accumbens astrocytes	(90) (88, 89) (94) (94)
KOP	mRNA	Human PBMC, whole blood cells Mouse splenocytes, T lymphocytes	(88, 89) (90)
NOP	Protein	Human PBMC	(88)

However, many other studies did detect MOP, DOP, and KOP receptors in immune cells of various species (Table 3). MOP receptor mRNA was identified by PCR in blood CD4^+^ T lymphocytes, monocytes/macrophages and granulocytes from healthy human donors, in PBMC and granulocytes from rhesus monkeys (96), and in rat peritoneal macrophages (98). Decreased levels of MOP receptor mRNA were found in blood lymphocytes of heroin addicts on methadone maintenance compared to healthy controls (97). In the latter study, similar results were obtained for DOP receptor mRNA (97). DOP receptor mRNA was additionally found in mouse splenocytes and enriched T lymphocyte fraction (90, 109), in lymph node dendritic cells and CD4^+^ T lymphocytes of mice immunized with ovalbumin (but not in non-immunized mice) (110), and in human blood lymphocytes (assessed by Northern blot) (91). Also KOP receptor mRNA was detected by PCR or Northern blot in blood lymphocytes (90, 91), monocytes (111), PBMC and CD4^+^ T lymphocytes from healthy human donors, and in PBMC from rhesus monkeys (113). In rheumatoid arthritis patients, KOP receptor mRNA was found in blood T and B lymphocytes, monocytes/macrophages and natural killer cells. Analysis of the whole blood cell samples revealed that the mRNA levels were lower in patients with high pain scores compared to those with less severe pain (112). For all three receptors, the mRNA transcripts were cloned and sequenced, and found to be nearly (98%) or completely (100%) homologous to the human, rat or mouse brain receptors (90, 91, 96, 98, 109, 113). Additionally, qPCR revealed mRNAs of MOP, DOP, and KOP receptors in blood leukocytes, and at higher levels in leukocytes (comprising macrophages, neutrophils and T lymphocytes) isolated from injured sciatic nerves in a mouse model of neuropathic pain. Activation of each receptor by selective agonists led to the secretion of opioid peptides from immune cells isolated from wild-type mice, but not from the MOP, DOP or KOP receptor knockout mice [(99); see also below]. This suggests that leukocyte MOP, DOP, and KOP receptors were functional and encoded by the same genes as neuronal receptors (99). NOP receptor mRNA was identified by PCR or qPCR in blood lymphocytes, monocytes and granulocytes from healthy human donors (88, 115, 116). The qPCR showed decreased NOP receptor mRNA levels in blood granulocytes from patients with sepsis (117), and comparable levels in blood eosinophils in patients with asthma and healthy volunteers (118). The sequencing of PCR products revealed that NOP receptor transcripts from immune cells and brain were identical (115, 116). Some studies detected the NOP receptor mRNA transcripts in leukocytes at levels comparable to those in human cerebral cortex (115), whereas others found them at very low amounts (88, 121). Together, opioid receptor mRNAs appear to be expressed at relatively low levels in immune cells as compared to neuronal tissue or opioid receptor-expressing immune cell lines (88–91, 109, 110, 121, 122). Additionally, opioid receptor mRNA levels in immune cells can be modified (elevated or diminished) by pathological conditions or pharmacological treatments *in vivo* (97, 99, 110, 112, 117). These issues need to be considered in order to obtain sufficient amount of tissue (leukocyte numbers) for the analysis.

**Table 3:** Expression of opioid receptors in native, not cultured immune and glial cells.

**Opioid receptor**	**mRNA, protein**	**Cell types**	**References**
MOP	mRNA	Human blood lymphocytes, monocytes/macrophages or granulocytes Rhesus monkey blood PBMC, granulocytes Rat peritoneal macrophages Mouse blood and injured nerve immune cells^*^ Rat nucleus accumbens, human brain, rat, mouse brain or spinal cord microglia	(96, 97) (96) (98) (99) (94, 100)
	Protein	Human blood lymphocytes, monocytes or granulocytes Rat splenocytes Rat, mouse brain or spinal cord microglia Mouse brain or spinal cord astrocytes Mouse brain oligodendrocytes	(101, 102) (103) [(100, 104)^#^, (105)^#^, (106)^#^] [(106)^#^, (107, 108)^#^] (108)^#^
DOP	mRNA	Human blood lymphocytes Mouse splenocytes, T lymphocytes Mouse lymph node dendritic cells, CD4^+^ T lymphocytes Mouse blood and injured nerve immune cells^*^	(91, 97) (90, 109) (110) (99)
	Protein	Human blood lymphocytes, monocytes or granulocytes Rat splenocytes Mouse brain astrocytes	(101, 102) (103) (108)^#^
		Mouse brain oligodendrocytes	(108)^#^
KOP	mRNA	Human blood lymphocytes, monocytes or natural killer cells Rhesus monkey blood PBMC Mouse blood and injured nerve immune cells^*^ Rat nucleus accumbens microglia Rat nucleus accumbens astrocytes	(90, 91, 111–113) (113) (99) (94) (94)
	Protein	Mouse peritoneal macrophages Mouse brain astrocytes Mouse brain oligodendrocytes	(114) (108)^#^ (108)^#^
NOP	mRNA	Human blood lymphocytes, monocytes, granulocytes or eosinophils Rat brain microglia Fetal human brain, adult and postnatal rat brain astrocytes	(88, 115–118) (119) (119, 120)
	Protein	Human blood granulocytes Fetal human brain, postnatal rat brain astrocytes	(116, 121) (120)^#^

* *Include monocytes/macrophages, neutrophils, and T lymphocytes*.

# *Indicates that the antibody staining specificity was not convincingly verified or not tested at all*.

Detection of MOP, DOP, and KOP receptor proteins is more challenging due to the low expression levels mentioned above and poor specificity of antibodies (123–125). Nevertheless, a few studies described stereospecific and high-affinity opioid binding sites in leukocytes (Table 3). The binding of radiolabeled naloxone displaceable by naltrexone was found in healthy human blood lymphocytes (97), although the receptor type is unclear, since these ligands do not distinguish MOP, DOP, and KOP receptors (Table 1). In contrast, binding of radiolabeled MOP receptor agonist (dihydromorphine) or of radiolabeled DOP receptor agonist (deltorphin I) suggested the presence of MOP receptors in monocytes and granulocytes, and of DOP receptors in granulocytes from blood of healthy human donors (101, 102). In rat splenocytes, MOP receptors were detected using radiolabeled agonist DAMGO whose binding was displaced by the antagonist CTAP, both MOP receptor selective ligands. Similarly, DOP receptors were identified using its selective ligands, the radiolabeled antagonist naltrindole whose binding was displaced by the agonist SNC80 (103). KOP receptor protein was found in mouse peritoneal macrophages by flow cytometry and fluorescently labeled KOP receptor agonist, and the labeling intensity was diminished by the selective antagonist norBNI (114) (see also Table 1 for ligands). NOP receptor protein detection appears variable, with high affinity radiolabeled N/OFQ binding in granulocytes (116), but lack of such binding in PBMC (88) from human blood. The latter research group recently detected NOP receptor binding (reversible by selective NOP receptor antagonist SB-612111) in human blood granulocytes using a novel fluorescent probe for the receptor (a red fluorophore-ATTO594 conjugated to the N/OFQ) (121).

In summary, all four MOP, DOP, KOP, and NOP receptors have been identified *ex vivo* at the mRNA and protein levels in various types of immune cells in humans, rhesus monkeys, rats and mice (Table 3).

### Glia

Compared to peripheral immune cells, the expression of opioid receptors in glia has been less examined, most studies focused on MOP receptors, and the findings are contradictory. MOP receptor mRNA was not detected in microglia and astrocytes in the spinal cord of rats chronically treated with vehicle or morphine, using *in situ* hybridization for MOP receptor mRNA combined with immunofluorescent staining of microglia and astrocyte markers (93). MOP receptor mRNA was also not found using qPCR in astrocytes isolated from nucleus accumbens of rats after acute injection with vehicle or morphine (94). It was also undetected by transcriptomic profiling of microglia from the spinal cord of naïve or morphine-treated mice (92). Additionally, double-immunofluorescence did not detect MOP receptor protein in spinal cord microglia and astrocytes following single application of vehicle or morphine in mice (95). Similarly, MOP receptor mRNA or protein were not found in spinal cord microglia of naïve transgenic mice with fluorescently labeled microglia (CX3CR1-eGFP) or with fluorescently tagged MOP receptors (MOP-mCherry) (92) (Table 2).

In contrast, several other studied have identified MOP receptors in human and rodent microglia (Table 3). Using qPCR, very low MOP receptor mRNA levels were found in microglia isolated from nucleus accumbens of rats acutely treated with vehicle or morphine (94). Transcriptomic analysis revealed MOP receptor mRNA in microglia in cerebral cortex of humans with no pain history, and in various brain areas and spinal cord of naïve mice or rats. Using immunofluorescence in transgenic mice expressing MOP receptors in microglia (CX3CR1-eGFP–MOP-mCherry), the percentage of MOP receptor-positive microglial cells ranged between 35 and 52% in brain, and between 37 and 42% in the spinal cord. The presence of MOP receptor protein in Golgi apparatus suggested that the receptors might be synthetized by microglia (100). Utilizing MOP-mCherry mice and double labeling in wild-type mice using astrocyte marker and MOP receptor antibodies (whose staining specificity was confirmed in MOP receptor knockout mice), MOP receptor protein was detected in astrocytes in various brain regions (107). Additional studies detected MOP receptor protein in naïve rat and mouse microglia or astrocytes in the spinal cord, or in mouse brain astrocytes and oligodendrocytes using antibody-based double labeling of the glial cell markers and MOP receptors, but the staining specificity of antibodies was not convincingly verified or not tested at all (104–106, 108).

Only a few studies assessed DOP, KOP, and NOP receptors in glia. No DOP receptor mRNA and low levels of KOP receptor mRNA were detected by qPCR in microglia and astrocytes isolated from nucleus accumbens of rats acutely injected with vehicle or morphine (94). NOP receptor mRNA was found in microglia and astrocytes in adult rat brain (119) or in astrocytes in rat brain until the third postnatal week, and in fetal human brain (120) by *in situ* hybridization for NOP receptor mRNA combined with immunofluorescent staining of microglia and astrocyte markers. Proteins of DOP, KOP, and NOP receptors were detected in astrocytes or oligodendrocytes in mouse or rat brain, but the staining specificity of antibodies was not unequivocally proven or not tested (108, 120).

Together, although there is still a controversy (Table 2), MOP receptors might be expressed in microglia and astrocytes, but more well-controlled studies are needed to verify the presence of DOP, KOP, and NOP receptors in native glia (Table 3).

## Modulation of Pain by Opioid Receptors in Immune and Glial Cells

### Immune Cells

A recent study expands the classical model of neuronal opioid receptor-mediated analgesia by showing the contribution of MOP, DOP, and KOP receptors in immune cells to the amelioration of pain (99). The activation of leukocyte opioid receptors led to the secretion of opioid peptides (β-endorphin, Met-enkephalin and dynorphin A 1-17), which subsequently acted at peripheral neuronal opioid receptors in injured tissue, and relieved pain (Figure 1B). Specifically, in a mouse model of the sciatic nerve injury, exogenous agonists selective at MOP (DAMGO), DOP (DPDPE), and KOP receptors (U50,488; Table 1) inhibited mechanical hypersensitivity following injection at the damaged nerve infiltrated by immune cells (neutrophils, macrophages, T lymphocytes). The analgesia was attenuated by opioid peptide antibodies injected at the injured nerve or by leukocyte depletion in wild-type mice. This effect was also diminished in mice lacking opioid peptides (β-endorphin-, PENK-, PDYN-knockout) compared to wild-type mice. This decrease in analgesia was restored by the transfer of wild-type, but not opioid receptor-lacking leukocytes (from MOP, DOP, or KOP receptor knockout mice). *Ex vivo*, exogenous opioids triggered the release of opioid peptides from immune cells isolated from damaged nerves of wild-type mice, measured by immunoassays. The release was dependent on Gαi/o and Gβγ proteins, phospholipase C (PLC), inositol 1,4,5-trisphosphate (IP_3_) receptors and intracellular Ca^2+^. This exogenous opioid-induced secretion of opioid peptides did not occur in immune cells isolated from nerves of mice lacking opioid peptides or receptors, which confirmed the specificity of opioid peptide antibodies used in immunoassays. Importantly, the opioid receptor-coupled intracellular Ca^2+^ pathway in immune cells mediated analgesia *in vivo* (99). Together, in addition to opioid receptors on peripheral sensory neurons [e.g., (99, 126–130)], analgesia can be mediated by MOP, DOP, and KOP receptors in immune cells (99). In contrast to the conventional action of neuronal opioid receptors (i.e., the inhibition of the release of pain-inducing mediators; analyzed in the introduction), analgesia mediated by leukocyte opioid receptors involves the secretion of pain-inhibiting opioid peptides (99). These effects may explain the enhanced analgesia of intra-articular morphine in patients with synovial tissue infiltrated by immune cells, following knee surgery (131). There are currently no data on the modulation of pain by leukocyte NOP receptors.

### Glia

The actions of opioids on glial cells are typically discussed in relation to analgesic tolerance and paradoxical hyperalgesia termed opioid-induced hyperalgesia (OIH). Analgesic tolerance represents a progressive decrease of analgesia with prolonged agonist use or the need to increase the agonist dose to maintain analgesia. The OIH is usually described as hypersensitivity to painful stimuli upon chronic opioid use [reviewed by (132, 133)]. Nevertheless, since the abrupt discontinuation of prolonged opioid use can result in a withdrawal syndrome, including enhanced pain, the OIH may in fact represent the opioid withdrawal-induced hyperalgesia [reviewed by (134)]. The majority of studies have focused on the effects of morphine and on MOP receptors, but only a few directly addressed the microglia following chronic morphine treatment, and showed that tolerance and/or OIH were reduced by microglia depletion (105, 135) (Figure 1C). The former study proposed that morphine activated MOP receptors on spinal cord microglia to increase the expression of purinergic P2X4 receptors, which triggered the (MOP receptor-independent) release of brain-derived neurotrophic factor (BDNF) from microglia. The secreted BDNF induced downregulation of the K^+^-Cl^−^ co-transporter KCC2 in GABAergic neurons, which resulted in their disinhibition. These actions were implied to mediate OIH, but not tolerance (105). Hayashi et al. (135) suggested that both tolerance and OIH involved microglia MOP receptor-induced secretion of arachidonic acid (AA) and subsequent activation of microglial large conductance Ca^2+^-activated K^+^ (BK) channels in the spinal cord. Several other studies showed the correlation between tolerance or OIH and the involvement of spinal cord microglia and/or astrocytes in morphine-treated animals. These observations were based on the increased glia numbers or elevated expression of nuclear factors and protein kinases in these glial cells (95, 136–138). Similar indirect effects were reported for other opioids acting at MOP receptors, including remifentanil, fentanyl and buprenorphine (139–141). Agonists of DOP and KOP receptors have not been tested. One study found elevated expression of astrocytes, but not microglia, in the spinal cord following N/OFQ treatment (142).

Nevertheless, it is unclear how opioids or N/OFQ would affect the glia, since the expression of opioid receptors in native glia is still debatable (see above), most of those studies did not directly examine the involvement of glial MOP or NOP receptors, OIH was observed in triple MOP, DOP, and KOP receptor knockout mice (143, 144), and the effects of morphine on toll-like receptor 4 in glia remain controversial [reviewed by (133)]. Furthermore, except for Chang et al. (141) who used postoperative pain model, all other studies exclusively tested naïve mice or rats. Additionally, opioids were used at unconventional ultra-low doses or high doses which exceeded the analgesic doses used in pathological pain models, they were sometimes injected repetitively every few minutes, and hyperalgesia was measured 12 h, 24 h or 4 days following the last dose, which indicates opioid withdrawal-induced hyperalgesia (95, 105, 135–142). Therefore, these effects can be viewed in the context of dependence rather than pain management, which is in line with clinical findings [reviewed by (134, 145)]. It is thus essential to examine actions of opioids on glia in models of pathological pain using analgesia-relevant paradigms. Accordingly, clinical studies suggested that OIH may not be a significant concern if opioids are used at regular doses in pathological pain conditions, and when their use is discontinued gradually (146, 147).

## Opioid Peptides and N/OFQ in Immune and Glial Cells

### Immune Cells

Immune cells also contain endogenous ligands of opioid receptors. The opioid peptide-containing leukocytes have been extensively investigated over the last decades [reviewed by (58, 148, 149)] and will only be briefly addressed here. Transcripts of POMC, PENK and PDYN, as well as enzymes required for POMC and PENK processing are expressed in T and B lymphocytes, macrophages or granulocytes in peripheral inflamed tissue in rats and mice (150–154). Consequently, the corresponding opioid peptides β-endorphin, enkephalins and dynorphin A 1-17 were detected in various immune cells from blood, lymph nodes or peripheral injured tissue in rats, mice and humans (99, 155–158). Opioid peptide-containing immune cells extravasate using adhesion molecules (P- and E-selectins, integrins α_4_ and β_2_, intercellular adhesion molecule-1) and chemokines (CXCL1, CXCL2/3) to accumulate in damaged tissue. Subsequently, the leukocytes secrete opioid peptides spontaneously or in response to stressful stimuli (experimental stress, surgery) and releasing agents, such as corticotropin-releasing factor, IL-1β, chemokines CXCL1, and CXCL2/3, noradrenaline, mycobacteria, and exogenous opioids. The released opioid peptides activate opioid receptors on peripheral sensory neurons and locally inhibit pain. Such analgesic actions have been demonstrated in rodent models of somatic and visceral inflammatory, neuropathic and bone cancer pain (99, 156, 159–170), as well as in patients with arthritis undergoing knee surgery (171–173). Notably, due to the continuous presence of immune cell-derived opioid peptides and enhanced MOP receptor recycling, the analgesic tolerance at peripheral MOP receptors in inflamed tissue is greatly reduced, in animals and humans (131, 174).

The mRNA of the N/OFQ precursor, ppN/OFQ, was detected in porcine splenocytes, blood granulocytes and eosinophils from healthy volunteers and patients with sepsis or asthma (117, 118, 175). Additionally, the ppN/OFQ mRNA was found in blood neutrophils, monocytes and lymphocytes from healthy donors, and neutrophils secreted the N/OFQ upon degranulation *ex vivo* (176). However, the functional relevance of immune cell-derived N/OFQ *in vivo* has not been elucidated.

### Glia

There are numerous studies reporting expression of opioid peptides or their precursors in cultured glia [reviewed by (177)], but very few examined native tissue. The laboratory of Wang suggested analgesic effects of spinal cord microglia-derived β-endorphin and dynorphin A in rat models of inflammatory, neuropathic and bone cancer pain. However, they examined opioids in cultured microglia or in the spinal cord homogenates (178–180). Only in one study, β-endorphin was shown in spinal cord microglia *ex vivo*, but the staining specificity of the antibody was not tested (179). Therefore, the conclusive data on the expression of opioid peptides in native glia and their contribution to pain inhibition are still needed. The mRNA and protein of PENK were detected in cerebellum astrocytes in young rats (181), whereas N/OFQ was found in astrocytes of postnatal rat and fetal human brains (120), suggesting their role in brain development. The PDYN mRNA and protein were shown in astrocytes in human cerebral cortex, but their biological significance was not addressed (182).

Collectively, there is convincing evidence of analgesic actions mediated by immune cell-derived opioid peptides in animal models and in humans. The role of immune cell-derived N/OFQ and glia-derived opioids and N/OFQ in the context of pain is yet to be elucidated.

## Conclusions

The classical opioid receptors MOP, DOP, and KOP, and their endogenous ligands opioid peptides are expressed in immune cells accumulating in peripheral inflamed tissue. Activation of all three leukocyte opioid receptors by exogenous opioids has been shown to release opioid peptides, which acted at peripheral neuronal receptors to diminish pain. This has wide clinical implications, since most painful conditions are associated with immune response, including inflammatory neuropathies, arthritis, cancer and postoperative pain. Therefore, the broad-spectrum inhibition of immune responses should be avoided, as this may exacerbate pain and diminish exogenous opioid analgesia. Furthermore, as these actions occur in peripheral tissue, the detrimental side effects resulting from the activation of MOP, DOP, and KOP receptors in the brain are precluded. It will be interesting to find out whether this can also apply to NOP receptors and N/OFQ. First, however, the involvement of immune cell NOP receptors and N/OFQ in pain modulation needs to be shown. The widely discussed OIH is often linked to the actions of opioids (mostly morphine) via glia (primarily microglia, but also astrocytes). Nevertheless, even if these effects are mediated (indirectly or directly) via glial MOP receptors, they seem to be related to dependence/withdrawal rather than pain treatment, in line with clinical findings. More well-controlled studies are needed to verify the presence of DOP, KOP, and NOP receptors, opioid peptides and N/OFQ in native glia, and to elucidate their role *in vivo*. Considering that there is strong evidence of discrepancies between *in vivo* and *in vitro* conditions, which is particularly relevant to the immune system, examination of native cells and tissue, without culturing, is preferable whenever possible. Factors that can influence the expression of the opioid system in immune and glial cells, and that may contribute to the inconsistencies among the studies include the examined cell population (e.g., lymphocytes, granulocytes, monocytes, macrophages, microglia or astrocytes), their subpopulations (e.g., T or B lymphocytes, T helper 1 or T helper 2 lymphocytes, M1 or M2 macrophages or microglia), the tissue they originate from, the *in vivo* physiological vs. pathological conditions, type and duration of the pathological state, and *in vivo* pharmacological treatments. Furthermore, methodological procedures need to be carefully designed, including protocols to obtain sufficient number of cells, verification of cell viability, the isolation techniques that enable high RNA and protein yields without contaminants (e.g., DNA contamination, remaining sample preparation reagents, excessive protein amounts), optimal design of PCR primers and probes for efficient qPCR, and the use of stringent controls to avoid false positive or negative results (e.g., no-RT controls for qPCR, antibody staining specificity controls for immunostaining) [(183); Celik and Machelska, submitted].

## Author Contributions

HM conceptualized and wrote the manuscript with the input from MC. MC prepared the figure. HM and MC approved the final version of the manuscript.

### Conflict of Interest

The authors declare that the research was conducted in the absence of any commercial or financial relationships that could be construed as a potential conflict of interest.
